# The affordances of clinical simulation immersive technology within healthcare education: a scoping review

**DOI:** 10.1007/s10055-022-00745-0

**Published:** 2023-01-14

**Authors:** Stephen Aiello, Thomas Cochrane, Charles Sevigny

**Affiliations:** 1 Department of Paramedicine, Auckland University of Technology, Auckland, New Zealand; 2 Centre for the Study of Higher Education, The University of Melbourne, Melbourne, VIC Australia; 3 Department of Anatomy and Physiology, Faculty of Medicine, Dentistry and Health Sciences, The University of Melbourne, Melbourne, VIC Australia

**Keywords:** Healthcare, Immersive reality, Clinical simulation, Stress, Education design

## Abstract

Whilst clinical simulation is established as an effective education tool within the healthcare community, the inability to offer authentic educational learning environments remains problematic. Advances in technology such as immersive virtual reality offer new opportunities to enhance traditional practice to an extent that may transform learning. However, with traditional clinical simulation stress and anxiety can both hinder performance and learning, yet it is unknown what nuances are applicable within a clinical virtual simulation environment. Determining potential benefits, drawbacks (including related stress and anxiety) and affordances of immersive technology clinical simulation designs may help provide an understanding of its usefulness. The aim of this scoping review is to investigate the range and nature of evidence associated with immersive virtual reality clinical simulation and education design. In addition, the review will describe authentic immersive technology clinical simulation use and reported stress response measurements. A search of seven electronic database and grey literature was performed in accordance with the Joanna Briggs Institute methodology. A key term search strategy was employed with five themes identified and investigated: (1) Healthcare professionals, (2) Clinical simulation, (3) Immersive virtual reality, (4) Stress/anxiety and (5) Authentic learning design. Application of the search strategy resulted in a hit total of 212 articles. Twelve articles met inclusion criteria. With most literature focusing on procedural performance and non-transferable education needs, there was a paucity of research that specifically investigated immersive virtual reality clinical simulation education and related stress. Therefore, this scoping review contributes new understandings by providing valuable insight and potential research gaps into current immersive virtual reality clinical simulation, its relationship to stress and the education design models currently being utilised to develop these concepts.

## Introduction

Authentic learning is rooted in constructivist theory, which advocates that actively engaging with problems and materials constitutes the best way to learn and teaches students *how to think* like a member of their own discipline (Meyers and Nulty 2009). Whilst direct patient contact is seen as the traditional opportunity to train Healthcare professionals (HCP) in practice, safety and competence, early education is often dominated by theoretical connections. Clinical safety requires preparedness, awareness of hazards, anticipation, resilience and flexibility (Vincent et al. 2010). Competence requires the development of sound clinical judgement and diagnostic reasoning (Simmons 2010). With this, and to add context to theory, clinical education in recent years has increasingly relied upon the use of clinical simulation education for safety and competence (O'Meara et al. 2015).

Traditional clinical simulation can be defined as “A technique that creates a situation or environment to allow persons to experience a representation of a real event for the purpose of practice, learning evaluation, testing, or to gain understanding of systems or human actions.” (Lioce 2020). Clinical simulation includes a range of low- and high-fidelity manikin-based models and role play (Mills et al. 2016). Fidelity itself can be described as “the degree to which the simulation replicates the real event…” (Lioce 2020). These definitions emphasise the connection to the varied environments that HCP work but within a controlled education space. However, the need to introduce educational innovation is still a pressing matter with the health education community constantly requiring methods that can be utilised to enhance and improve the education process (Crisp et al. 2008). The push towards innovation is driven by limited laboratory space that fails to accommodate increased student numbers and increased cost for equipment such as high fidelity manikins and skill related consumables (Fealy et al. 2019). This in addition to advances in technology mean that new opportunities may now exist that offer a cost and labour effective approach to HCP education.

### Background

The close contact environment required for clinical simulation education has been both problematic and challenging due to the COVID-19 pandemic. Traditional simulation education requires both potential victims and carriers of the virus to interact within a small room. Whilst mandatory testing has been implemented in most institutions, the risk of contracting the virus increases when multiple people come together in a small space (Ahmed et al. 2020). COVID-19 has changed the educational landscape for many, and it is therefore necessary to explore and potentially mitigate the impact of the virus by providing a transformative learning experience. With this, education facilities across the world are enacting contingencies that will allow HCP’s the opportunity to continue to learn whilst keeping learners safe and yet still provide an engaging and supportive educational experience (Brown et al. 2020). One such intervention is the use of virtual technology in education.

Virtual technology advances include augmented reality (AR), mixed reality (MR/XR) and immersive virtual reality (IVR) to name but a few. Whilst each technology differs slightly, the overarching attributes include layers of sensory information that offer the user a simulated experience of sound, sight, smell and touch similar to the physical world. Users are typically immersed within environments that offer real-world situations and/or representations of virtual humans. This now offers a potential to interact and explore authentic environments in a 360-degree visual format and transcend the boundaries of the physical world (Cochrane et al. 2020). The introduction of IVR provides an opportunity for technology to play a central role in the learning experience and may improve knowledge and cognitive skill when compared to traditional education (Kyaw et al. 2019). The goal of IVR is to change the perception of the user to that of being physically present (immersed) within a non-physical world. The challenge, however, in creating connections between concepts covered in theory and real-world phenomena can at times feel forced or awkward (Stein et al. 2004).

Authentic IVR learning practice facilitates learning through the development and initiation of student-centred authentic tasks (Cochrane et al. 2020). This involves critical inquiry, problem solving and meaningful real-world outcomes that lead to the construction of knowledge. Within an authentic context, clinical simulation should offer a similar experience to those undertaken by real-world clinicians with educators able to craft the experience to resonate the “situated (lived) experience.” (Stein et al. 2004, p. 240). However, whilst exposing a student to a clinical environment, there should also be protection from the dangers and nonessential influences of the real-world (Cochrane et al. 2020). When IVR is complemented by manikin-based clinical simulationm an opportunity exists to provide a more meaningful learning experience whilst developing safety and competence (Wright 2004). Learning, however, is often aligned with learner emotional stress as they move beyond the bounds of their prior knowledge with new and unknown environments (Hase and Kenyon 2007). Here caution must be held with reports of novice clinicians experiencing high levels of cognitive overload and stress resulting in reduced performance (Mills et al. 2016).

IVR simulation-based educational technology does, however, have limitations. Scalability, system cost (Kavanagh et al. 2017), usability, motion sickness, insufficient realism (Kavanagh et al. 2017; McCloy and Stone 2001), lack of VR haptic feedback (Kavanagh et al. 2017) and ever-changing technological advances have historically challenged researchers and education providers when seeking to determine the potential viability (Wier et al. 2017). The technology also requires careful consideration to ensure that the intervention is fit for purpose and not contrived towards technology for the sake of technology (Cowling and Birt 2018; Kavanagh et al. 2017). With the cost of IVR technology falling in recent years, this now provides opportunity to employ a low-cost approach towards an authentic IVR learning experience (Papara et al. 2020). In addition, there is potential for hundreds of HCP’s per day to utilise the IVR technology regardless of their location (Fabris et al. 2019). Within a climate hindered by COVID-19, this approach may provide an enhanced educational experience without being cost prohibitive, limited by student numbers or student location.

### Rationale

Educators need to show creativity and innovation to help challenge learners to develop a wide range of meta-cognitive and cognitive skills when trying to establish deeper understanding within the safety of a clinical simulation environment. The concepts of heutagogy help theorise an education framework that lends itself to equip learners with appropriate skills for a lifetime of learning (Blaschke 2018). In the context of this paper, this reflects first understanding learner capabilities for problem solving, clinical diagnosis and the underlaying principles of learner agency and reflection within the IVR simulation environments (Hase and Kenyon 2007). An understanding of heutagogical technology approaches may therefore help with the future design of authentic student-determined learning and lend itself to safe clinical practice.

Clinical simulation offers a contextual educational experience that often fails to measure emotion and experience during traditional manikin based training. Moreover, with the introduction of innovative technology such as IVR into clinical simulation, it is uncertain as to the extent of stress and learning within an authentic IVR environment. It is therefore important to identify IVR environment simulation approaches that are redesigned for an enhanced learning experience rather than being contrived towards technology and replication of a behaviouristic learning design. Although recognised when investigated separately, there have been no scoping reviews detailing a triangulation of quantitative, qualitative and biometric feedback indicators that evaluate and assess (1) All Healthcare professionals, (2) Clinical simulation, (3) IVR, (4) Stress and (5) Authentic learning design. A scoping review of how immersive technology relates to traditional clinical simulation within health care practice will help to define key concepts, map existing research and importantly identify future research gaps within this emerging area of research.

### Objective

The relationship between immersive technology and what impact healthcare clinical simulation has on stress and education is uncertain. To help identify existing gaps in knowledge, the aim of this scoping review is to systematically investigate the range and nature of evidence associated with immersive virtual reality clinical simulation and education design. In addition, the review will describe authentic immersive technology clinical simulation education design and reported stress measurements.

## Methods

The review was conducted in accordance with the Joanna Briggs Institute (Population, Concept, Context) methodology for scoping review (Peters et al. 2020). An a priori was adhered to and aligned to Preferred Reporting Items for Systematic Reviews and Meta-Analysis extension for Scoping Reviews (PRISMA-ScR) framework (Tricco et al. 2018). The search was further guided by a three-stage approach as recommended by Peters et al. (2020). The scoping review protocol was registered prospectively with the Open Science Framework (OSF) on 20th October 2021: (https://doi.org/10.17605/OSF.IO/ZP7EC) (Aiello 2021).

### Eligibility criteria

Inclusion criteria (Table 1) and equivalent terms found in Appendix 1 were considered for review. Authenticity aligns to the Substitution, Augmentation, Modification, and Redefinition (SAMR) framework model. Papers that used IVR as a direct skill Substitute or Augmentation (with no functional change) were excluded as this does not meet the authentic conceptual framework of this investigation. This categorises the integration of technology within education as authentic if the creation or design transforms by Modification or Redefinition the learning activity (Wahyuni et al. 2020). Modification relates to a significant task redesign with Redefinition being the creation of new tasks previously seen as inconceivable.

**Table 1:** Inclusion and exclusion criteria

Inclusion criteria*	Exclusion criteria
Related to healthcare professionals	Languages other than English
Authentic use/testing of immersive technology	Animal or child studies
Clinical simulation	Guides/product & opinion reviews/ reports/patents
Stress response and measurement	Not Immersive technology and clinical simulation
Studies that relate to education design	IVR as a direct substitute with no functional change

*Full text only

Due to the large number of irrelevant studies from veterinary science and paediatric healthcare, a ‘human/humans’ and ‘adult’ limiter was applied. In addition, to ensure a quality approach non-primary research such as guides, product reviews, reports and opinion papers were excluded. Databases were searched from the earliest available date to the 1st November 2021 and included for pragmatic reasons only those of English language with full text. Finally, there are no cultural, geographical or gender-based exclusion interests in this setting.

### Identifying the research question

All supporting evidence that meets a Population, Concept, Context (PCC) structure was considered for inclusion. The following research questions were formulated within the PCC framework.

- *Question:* What is the association between immersive virtual reality technology (Concept), authentic traditional clinical simulation (Context) and healthcare training (Population)?
- *Sub-Question 1*: How is immersive virtual reality clinical simulation participant stress response measured?
- *Sub-Question 2:* Is authentic clinical simulation healthcare education supported by learning design?

Participants include qualified clinical personnel or students studying towards a clinical qualification. Those who did not include immersive technology and clinical simulation were excluded. In line with the broad inclusion criteria for a scoping review, alternate technology-based simulation concepts illustrating an authentic clinical learning environment were investigated. This further included psychometric and physiological stress measurement within immersive technology but excluded studies that used technology as a direct substitute without functional change.

### Information sources

A preliminary search of MEDLINE, the Cochrane Database of Systematic Reviews and JBI Evidence Synthesis was conducted and no current or underway systematic reviews or scoping reviews on the topic were identified.

This review considered both quasi-experimental and experimental study designs including non-randomized controlled trials, randomized controlled trials, before and after studies and interrupted time-series studies. The types of publications included full-text journal articles and Grey literature: Google scholar, books, theses, conference and symposium presentations. All publications of qualitative, quantitative and mixed-method primary research study types were included.

### Search strategy

To identify appropriate index terms and keywords, the search strategy was tested in two electronic databases MEDLINE (PubMed) and CINAHL (EBSCO). The author consulted with an experienced librarian and performed a comprehensive search using the title and abstract to identify the full strategy keywords and index terms. The final search strategy for all identified keywords and index terms was adapted for each included database and/or information source. Seven databases were searched on 1st November 2021 and included: ERIC, AMED, PsychINFO (OVID), CHINAHL, MEDLINE (EBSCO), SCOPUS and Web of Science (Appendix 2). Grey literature was searched in Google Scholar, and a review of included source reference lists for similar topics were investigated to identify relevant studies.

### Source of evidence selection

All relevant citation data were exported from databases to Endnote™ X9 (Clarivate Analytics, PA, USA) before being forwarded to Covidence™ (Veritas Health Innovation, Melbourne, Australia) for peer review. The software enabled the identification of duplicate study results, screening and data extraction. This process follows the Preferred Reporting Items for Systematic Reviews and Meta-Analyses (PRISMA-ScR) guidelines (Tricco et al. 2018).

For consistency and to determine any risk of bias, the study selection followed a three-step process. (1) Prior pilot testing of the source selection was performed to help refine eligibility criteria and discuss discrepancies. (2) Two independent reviewers (SA and TC) screened titles and abstracts against the inclusion and exclusion criteria followed by (3) a full-text screen. Disagreement between SA and TC was resolved by discussion and consensus or by a third reviewer (CS). The two reviewers (SA and TC) critically appraised the included studies within the Covidence™ customised fields. The Inter-rater reliability was 90% with a Cohen’s Kappa of 0.61.

### Data extraction and charting

A standardised data extraction tool was developed a priori in Microsoft Excel 2019 (Microsoft Corp., Richmond, WA, USA). Two independent reviewers (SA and TC) tested and refined the tool and then extracted the final eligible study data from Covidence™ into the predetermined fields. The data included specific details about the participants, concept, context, study methods and included:

- Study characteristics
- Participant characteristics
- Psychometric and physiological (Stress) measurement
- Immersive technologies
- Clinical simulation characteristics
- Education/learning design
- Outcome characteristics
- Key findings

Charting of the data was recorded independently by SA and TC with answers and detail later verified for consistency. Any disagreement between SA and TC was resolved through (1) discussion, (2) an additional review or (3) a third reviewer (CS). No data within the eligible studies required further clarification from the author. When we encountered duplicate reporting of the same research study, we selected one report (the most detailed) for full review.

### Synthesis of results

The findings of the final review are presented below in a tabular form with narrative synthesis. The narrative will explore the patterns, similarities and relationships to stress and authentic immersive simulation learning within healthcare education. The mapping of the results is reported by providing a visual representation of the data to support breadth, extent and range of activity that aligns to the scoping review objective. Synthesis of data was undertaken by the principal investigator (SA) with validation of findings discussed amongst the review team (TC and CS).

## Results

### Selection of sources of evidence

**Fig. 1:**
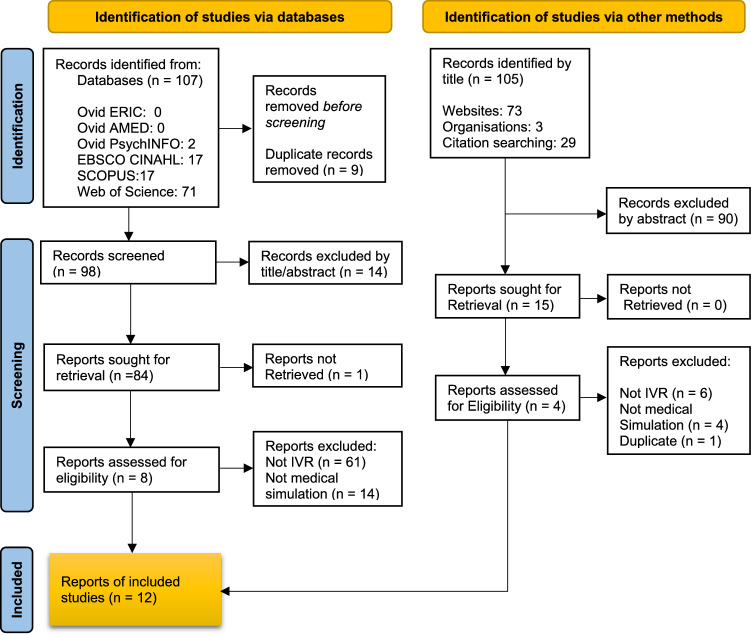
PRISMA-ScR flow chart of eligible studies. *Note* *IVR* Immersive virtual reality. From Page et al. (2021).

The search strategy identified 212 articles (107 database and 105 grey literature). Of these, 104 were excluded by title and/or abstract, 67 did not relate to IVR, 18 did not report healthcare clinical simulation, there were 10 duplicates and 1 study was excluded because we were unable to retrieve it. The remaining 12 articles were deemed eligible for inclusion and subject to this review (Fig. 1).

### Characteristics of sources of evidence

For the included cases, a summary of the publication year, setting, Journal, design and participant numbers is provided in Appendix 3. Studies were published in nine journals: with two each in “Research in Learning Technology”, “The Journal of Society for Simulation in Healthcare” and “Journal of Medical Internet Research” (JMIR), and one each in “Australian Journal of Education Technology”, “Multidisciplinary Digital Publishing Institute (MDPI); Information”, “Institute of Electrical and Electronic Engineers (IEEE)”, “Nurse Education Today”, “Computers, Informatics, Nursing (CIN)” and “Clinical Simulation in Nursing”.

**Fig. 2:**
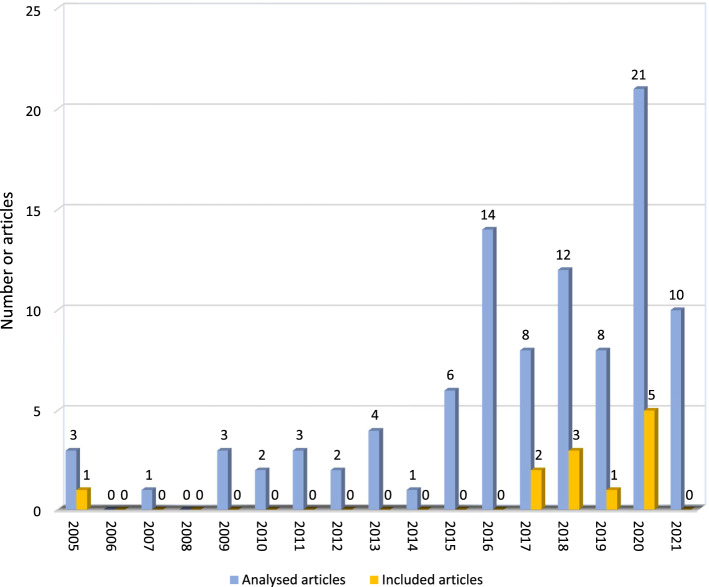
The number of analysed articles versus included articles by Year

The research was largely recent, with publications spanning between the 2005 and 2020 period (Fig. 2). The USA accounted for three studies (Chang et al. 2019; Johnsen et al. 2005; Wier et al. 2017), with both New Zealand (Aguayo et al. 2018; Cochrane et al. 2020) and Australia (Birt et al. 2017; Cowling and Birt 2018) two, and one each from Canada (Concannon et al. 2020), Germany (Lerner et al. 2020), Spain (Price et al. 2018), The United Kingdom (Rushton et al. 2020) and Belgium (Servotte et al. 2020) (Appendix 3).

There was a diverse study design with mixed methods being most common (Aguayo et al. 2018; Chang et al. 2019; Cochrane et al. 2020; Concannon et al. 2020; Johnsen et al. 2005; Price et al. 2018; Rushton et al. 2020; Servotte et al. 2020) followed by prospective cohort studies (Birt et al. 2017; Cowling and Birt 2018; Wier et al. 2017) and one feasibility study (Lerner et al. 2020) (Appendix 3).

### Synthesis of results in relation to the research questions

*Population* (Healthcare professional): The majority of articles sampled Paramedic (Aguayo et al. 2018; Cochrane et al. 2020; Cowling and Birt 2018) and Nursing (Price et al. 2018; Rushton et al. 2020; Servotte et al. 2020) undergraduate students. Qualified medical doctors accounted for two samples (Chang et al. 2019; Lerner et al. 2020), with one that included medical students (Johnsen et al. 2005). The remainder were occupational therapy students (Concannon et al. 2020), qualified paramedics (Birt et al. 2017) and a military team that included ‘army medics, doctors and nurses’ (Wier et al. 2017) (Appendix 4).

*Context* (Clinical Simulation): Virtual clinical simulation techniques included avatars (Birt et al. 2017; Concannon et al. 2020; Lerner et al. 2020; Servotte et al. 2020) and images of real people (Chang et al. 2019; Price et al. 2018). Manikin-based simulation (Aguayo et al. 2018; Chang et al. 2019; Cochrane et al. 2020; Price et al. 2018; Rushton et al. 2020; Wier et al. 2017) and augmented skill simulation were also investigated (Johnsen et al. 2005) (Appendix 4).

*Concept* (Immersive Technology): The technology related to alternate virtual electronic environments utilised multiple or single applications and included augmented virtual reality (Birt et al. 2017; Chang et al. 2019; Concannon et al. 2020; Cowling and Birt 2018; Lerner et al. 2020; Servotte et al. 2020), Immersive caves (Aguayo et al. 2018; Cochrane et al. 2020; Johnston et al. 2005; Rushton et al. 2020; Wier et al. 2017) and 360-degree virtual scenes (Aguayo et al. 2018; Cochrane et al. 2020; Price et al. 2018) (Appendix 4).

*Sub-Question 1* (Stress measurement): The assessment methods to investigate anxiety/stress were heterogenous in nature and included a range of quantitative, qualitative and biometric instruments. The range and diversity of questionnaire methods included the NASA Task load index (Chang et al. 2019), State-Trait Anxiety Inventory—Y1 / Test Anxiety Inventory (Concannon et al. 2020), Visual Analogue Scale / Mental Readiness Form (Servotte et al. 2020), Confidence Tool (Rushton et al. 2020) and unclassified questionnaires (Aguayo et al. 2018; Cochrane et al. 2020; Servotte et al. 2020). Wier et al. (2017) had one ancillary question related to a sense of “urgency and stressors”, but this did not form a main method. Birt et al. (2017), Cowling and Birt (2018), Johnsen et al. (2005) and Lerner et al. (2020) did not investigate stress or anxiety (Appendix 4).

Biometric/physiological testing was performed mostly by heart rate (Aguayo et al. 2018; Chang et al. 2019; Cochrane et al. 2020; Price et al. 2018) and skin conductance (Aguayo et al. 2018; Cochrane et al. 2020). Additional testing methods included an expression pedal (Aguayo et al. 2018), hexoskin suit / salivary cortisol (Chang et al. 2019), salivary alpha-amylase and blood pressure (Price et al. 2018). The remaining seven studies did not investigate biometric data (Birt et al. 2017; Concannon et al. 2020; Johnsen et al. 2005; Lerner et al. 2020; Rushton et al. 2020; Servotte, et al. 2020; Wier et al. 2017) (Appendix 4). Of the twelve results, only four mapped both subjective (questionnaire focus) and objective (Biometric / physiological) data together (Aguayo et al. 2018; Chang et al. 2019; Cochrane et al. 2020; Price et al. 2018).

*Sub-Question 2* (Authentic learning design): Whilst it is acknowledged that education and learning is implied within many of the included articles, only four were explicit in describing an educational design approach. Of note, all four articles described a Design Based Research methodology when describing their work (Aguayo et al. 2018; Birt et al. 2017; Cochrane et al. 2020; Cowling and Birt 2018) (Appendix 4).

**Fig. 3:**
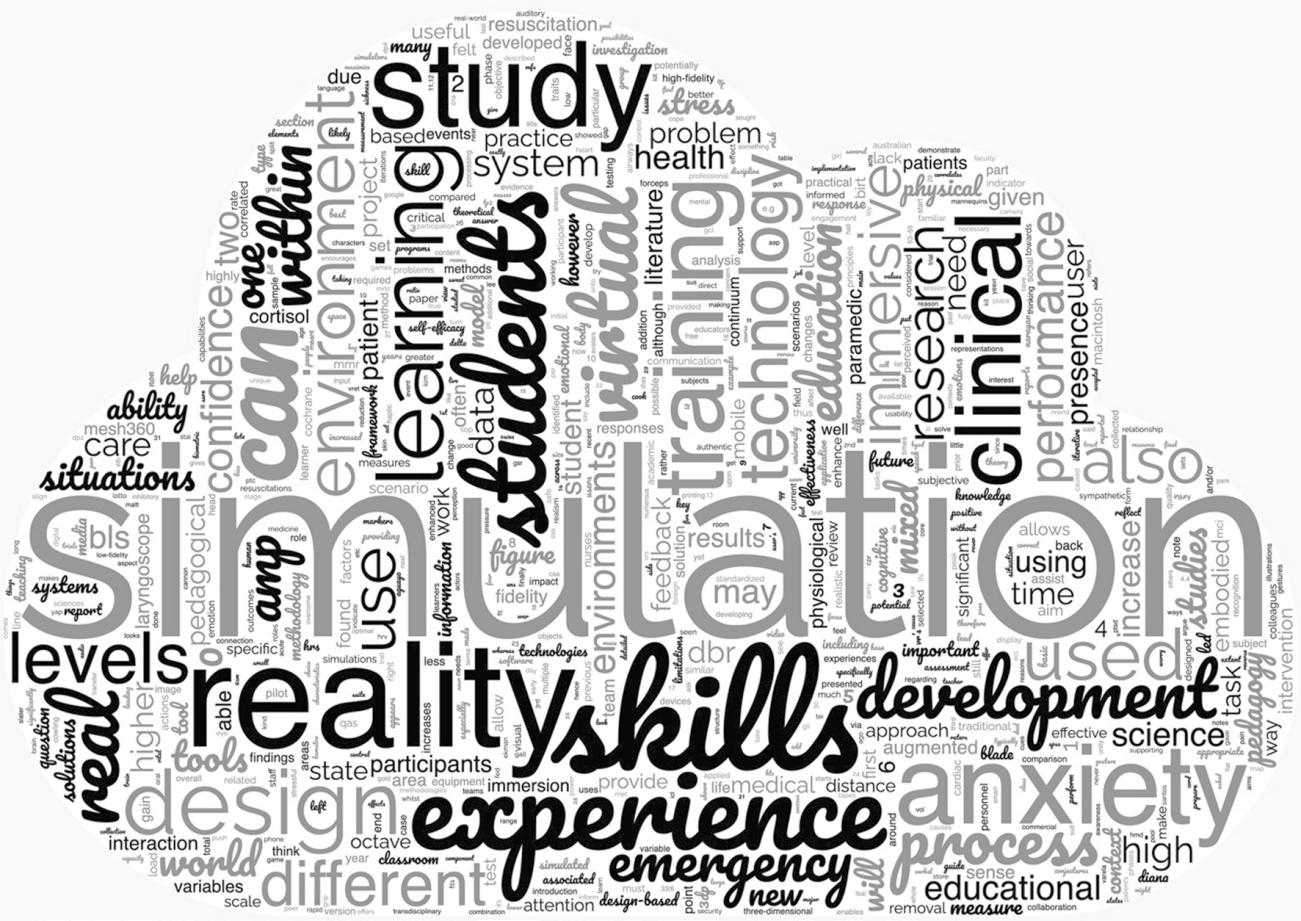
Word cloud: Term synthesis of included articles

A word cloud synthesis of commonly used terminology within the 12 articles is displayed, with frequency of use corresponding to the size of the term (Fig. 3).

## Discussion

### Summary of evidence

Our findings indicate a paucity of research given the wide date range inclusion criteria with most pertaining to 2017 onwards and reflect the infancy of this topic. Similar to the findings by Kyaw et al. (2019), there was also a distinct lack of research from low or middle income countries, which diminishes the pertinence of those that may be most in need of innovative learning strategies. However, unlike Kyaw et al. (2019), this review found many papers associated with allied health care professionals and adds to our understanding of the literature (Appendix 4).

**Fig. 4:**
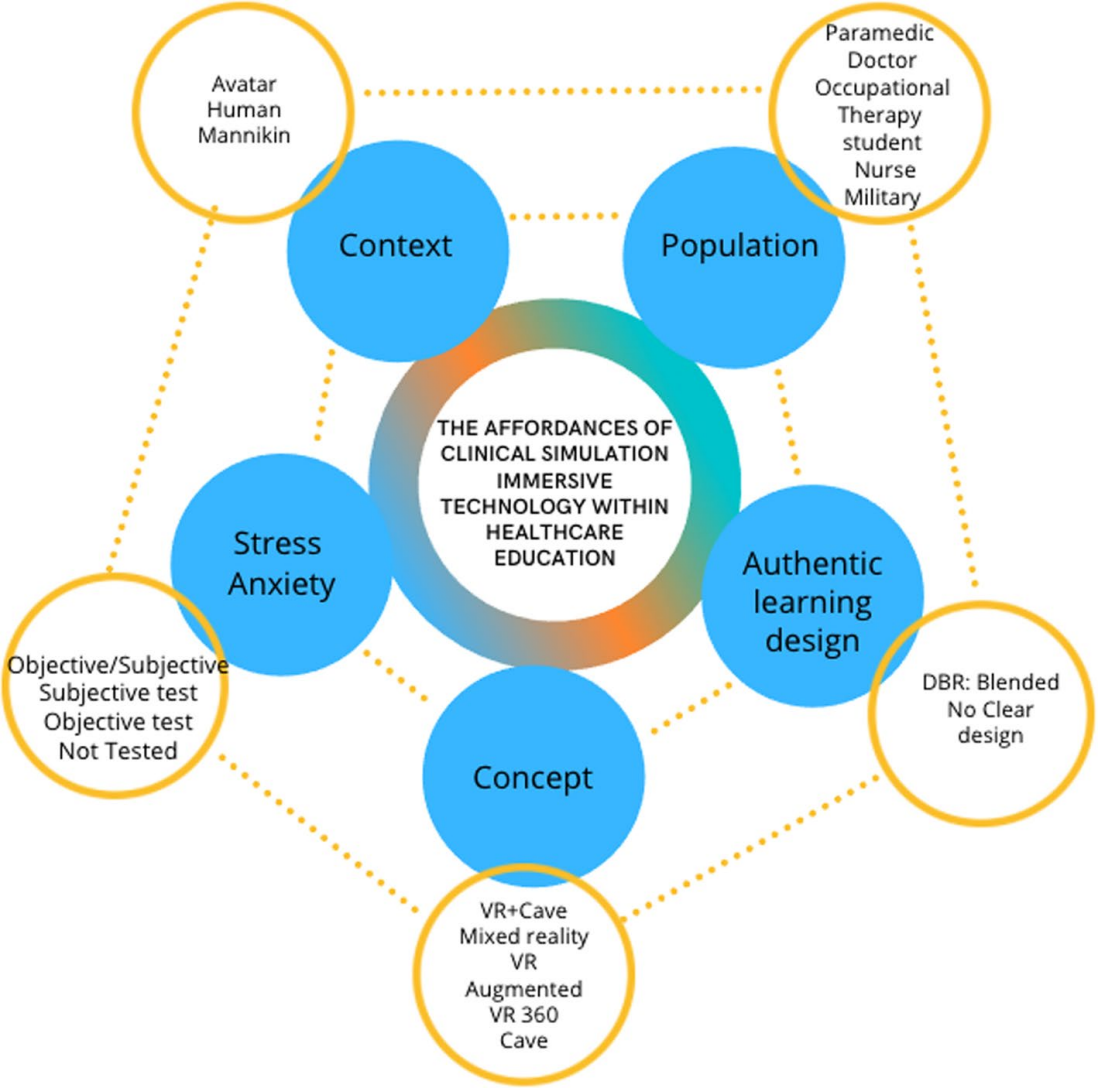
Relational diagram of themes

With technological advances, it is important to understand the association between immersive virtual reality technology (Concept), authentic traditional clinical simulation (Context) and healthcare training (Population). In addition, stress measurement tools and authentic learning designs themes are reported (sub-questions 1 and 2). This scoping review identified 12 studies that addressed these topics: Appendix 4 summarises the article themes and highlights the PCC question, sub-questions, context and outcome/findings. To our knowledge, this is the first review of the literature that explores the five related themes: (1) All Healthcare professionals, (2) Clinical simulation, (3) IVR, (4) Stress and (5) Authentic learning design (Fig. 4).

### The research question

Revisiting the research question, the heterogeneity found within the five themes shows that the current adoption of immersive technology within clinical simulation education is both widespread and desultory. It is evident that healthcare clinical simulation (first and second themes) is being utilised by a range of healthcare disciplines. It is clear that the integration of technology not only relates to the assessment of competency but also to support distance learning, reduce costs and promote effective learning (Birt et al. 2017). A possible explanation for this may be the rapid growth of clinical expectations for quality care within a safe yet efficient healthcare environment.

This scoping review identified several immersive technologies within healthcare simulation. These can be broadly categorised into three main areas: (1) those that performed traditional clinical simulation within an immersive (screen projected) CAVE environment and without being tethered to a computer (Johnsen et al. 2005; Rushton et al. 2020; Wier et al. 2017); (2) those able to view the IVR simulation within a 110–180 degree HMD environment (Birt et al. 2017; Concannon et al. 2020; Cowling and Birt 2018); (3) those who blended traditional simulation with a IVR 360 degree HDM environment (Aguayo et al. 2018; Chang et al. 2019; Cochrane et al. 2020; Learner et al. 2020; Price et al. 2018; Servotte et al. 2020).

A mixed modality of traditional manikin and an immersive cave design provides a virtual environment and context to simulation (Aguayo et al. 2018; Cochrane et al. 2020; Wier et al. 2017; Rushton et al. 2020). The benefit to this is that the HCP is provided with increased engagement by supporting kinaesthetic (hands-on) learning in conjunction with the stimulation of auditory, visual and haptic senses for a real-world experience. The remaining studies described virtual patients/avatars without haptic cue. The implications are exposing the HCP to an experiential learning and clinical decision making environment but without the tactile interaction found in real life (Servotte et al. 2020). The result is that a reduced level of feedback may negatively impact the HCP by detracting from the learning experience (Kavanagh et al. 2017).

Whilst there are advantages and disadvantages to each media, all allowed the user immersion and a sense of presence. Whilst the literature did detail the media quality, there was no discussion regarding fidelity standards. Limitations highlighted by Kavanagh et al. (2017) suggest system fidelity may be an important factor with lower latency and higher resolution media required to enhance realism. For many, a mixed modality approach was further enhanced by auditory stimulation such as screams and explosions (Cochrane et al. 2020; Chang et al. 2019; Price et al. 2018; Rushton et al. 2020; Servotte et al. 2020; Wier et al. 2017), artificial intelligent speech recognition (Concannon et al. 2020; Johnsen et al. 2005), haptic feedback (Birt et al. 2017), olfactory stimulus (smells) and distracting elements such as real smoke (Wier et al. 2017). It is of note that each stimulus is in the extreme and designed to invoke stress and a sense of emergence.

There are some concerns that virtual patients should be used only as a complimentary pedagogical resource and not replace human patient contact (Price et al. 2018). In addition, virtual patients may be a barrier to learning (King et al. 2018) and promote reduced empathy (Edelbring et al. 2011). IVR may also prove problematic when technological motivation becomes the drive rather than learning needs (Cowling and Birt 2018) or a novelty that may diminish (Kavanagh et al. 2017). However, when compared to human patients, Price et al. (2018) reported IVR to be as efficient as human assessment and offers insight into the potential learners experience and acceptance of realism when performing the task. Regardless of method, the constructivist approach posits that all offer the learner a range of clinical conditions and/or environments which would otherwise be rare or non-accessible.

### Sub-question 1

Sub-question 1 sought to determine how immersive virtual reality clinical simulation participant stress response is measured. The results show that the tools and measures used to investigate this relationship where categorised into three main areas: (1) those that did not measure stress or anxiety (Birt et al.^2017^; Cowling and Birt 2018; Johnsen et al. 2005; Lerner et al. 2020); (2) those that used subjective methods (Concannon et al. 2020; Rushton et al. 2020; Servotte et al. 2020; Weir et al. 2017); (3) those who used biometric/physiological (quantitative) tests (Aguayo et al. 2018; Chang et al. 2019; Cochrane et al. 2020; Price et al. 2018).

The differing self-reporting techniques used to determine and validate the subjective nature of IVR simulation stress and anxiety highlights a gap in our understanding for gold standard practice. Methods differed with simulation pre-brief provided by some to ensure psychological safety prior to testing (Learner et al. 2020; Servotte et al. 2020) followed by debrief to help understand the experiential emotion (Servotte et al. 2020; Weir et al. 2017). A concern with this is that most study interventions dealt with emergency events or actions which by default are uncertain and difficult to anticipate. Whilst pre-brief served to protect the participant, there is a potential consequence that the method may remove elements of test interest (stress/anxiety) by offering prior certainty and context to the experiment. This is highlighted by Servotte et al. (2020) where participant feedback requested enhanced pre-brief sessions and is in stark contrast to those who did not have a pre-brief and resulted in heightened pre-test anxiety (Concannon et al. 2020).

Virtual Reality Exposure Therapy (VRET) relates to those subjected to situational contexts that induce anxiety. The required outcome of VRET is to develop stress inoculation to a context or event without negatively impacting critical thinking or performance (Concannon et al. 2020). With this, Wier et al. (2017) suggests that by increasing the stress level there is greater opportunity to adapt. Adaptation towards the fear is theorised to occur when anxiety suppression is achieved and is known as inhibitory learning (Concannon et al. 2020). A possible explanation for its use within clinical simulation might be that it is designed to evoke emotion whilst helping to prepare for real-world clinical practice. That said, there is a question as to how do we safely provide stress, without causing harm?

None of the articles found within this review reported causing harm towards its participants, but Biometric data suggests the IVR simulation did increase the participant physiological response related to stress and anxiety (Aguayo et al. 2018; Chang et al. 2019; Cochrane et al. 2020; Price et al. 2018). Whilst it is unknown if this data relates towards the IVR technology, the hardware or the task at hand, it is somewhat surprising to find that the heart rate increased *prior* to simulation start and then reduced in rate *during* the simulation (Aguayo et al. 2018; Cochrane et al. 2020). An important observation is that the symptoms of pre-test anxiety also correlate with the qualitative findings of Concannon et al. (2020), Rushton et al. (2020) and Servotte et al. (2020) where the pre-clinical simulation phase was seen as “daunting…” with participants indicating a “…fear of the unknown.”(Rushton et al. 2020).

Pre-test anxiety and nervousness to some degree is to be expected and is a normal physiological and biological anticipatory response. However, stress can negatively impact skill performance and critical thinking (Wier et al. 2017) with potential for a loss of confidence and an inability to act (Rushton et al. 2020). Anecdotal findings by Aguayo et al. (2018) and Concannon et al. (2020) note that without instruction, participants performed (self-calming) diaphragmatic breathing techniques to potentially help mitigate and manage stress. The technique of cyclic inhalation and exhalation deep breathing lowers heart rate, blood pressure and sympathetic response and may assist with learner focus (Hopper et al. 2018).

It is possible that the use of IVR may mitigate anxiety with Price et al. (2018) reporting IVR to be as accurate for patient triage; 87.2% (SD = 7.2) versus 88.3% (SD = 9.65), but with lower levels of physiological stress measured in participant alpha-amylase, heart rate and blood pressure when compared to non-IVR triage simulation. This finding is consistent with Rushton et al. (2020) who described that IVR helped to develop coping skills and Concannon et al. (2020) and Chang et al. (2019) who observed reduced anxiety for the IVR group when compared to the non-IVR group. Overall there seems to be a gap in knowledge with limited investigation of pre-simulation anxiety and coping techniques. This is an important consideration for participant safety and is a topic for future research.

### Sub-question 2

Is authentic clinical simulation healthcare education supported by learning design? Similar to the findings of Kavanagh et al. (2017) and Kyaw et al. (2019), this study found limited explicit pedagogical detail underpinning a learning or education design within the articles. Whilst it could be implied that all the reviewed articles have a scientific education lean, four papers proposed Design Based Research (DBR) and described a pedagogical approach that met the SAMR model for authentic learning design (Aguayo et al. 2018; Birt et al. 2017; Cochrane et al. 2020; Cowling and Birt 2018). It is likely that whilst DBR is not commonplace within the wider literature it may offer a more agile and flexible approach to ever-changing technology and the needs of the healthcare workforce.

**Fig. 5:**
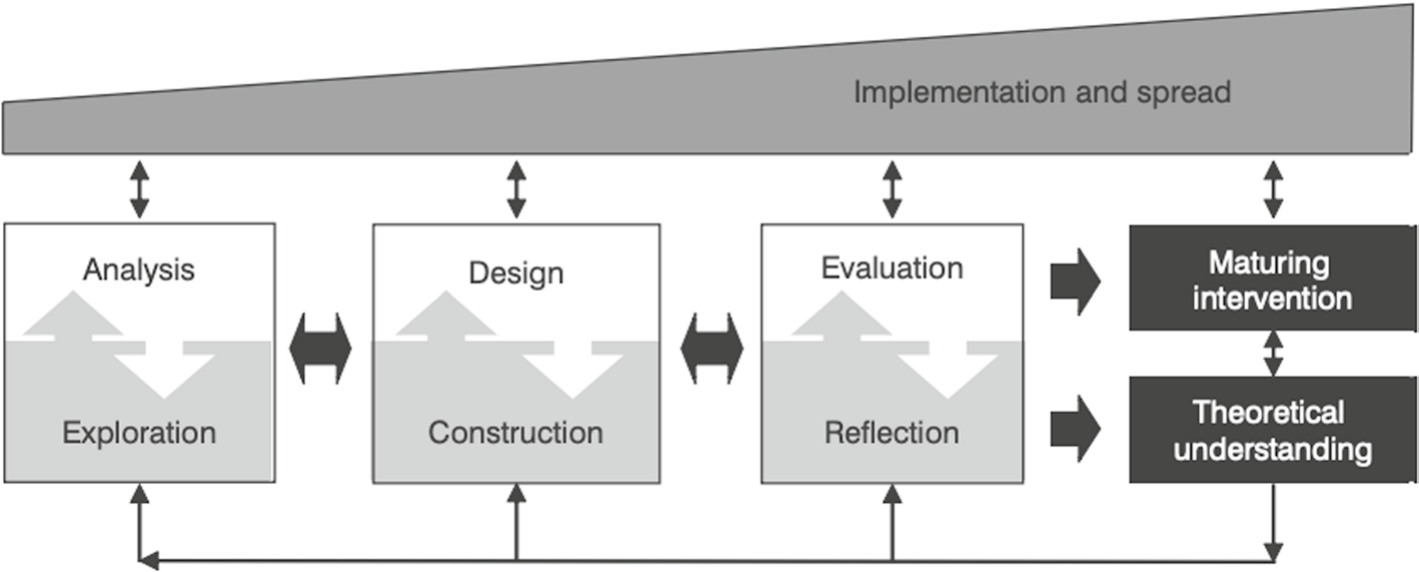
Design based research model. *Note* Generic model for conducting design research in education. From McKenney and Reeves (2012)

The principles of DBR require an iterative approach to technology and education based on learning outcomes. The approach requires finding solutions to an issue and investigating if the use of technology can help solve the problem (Amiel and Reeves 2008). This further requires constant feedback loops between theory, testing, applied practice and reflection whilst being flexible to adapt to newer technologies, methodologies, theoretical knowledge, and contextual challenge (Fig. 5). In broad terms, this allows DBR to leverage pedagogies that are informed by learning theories of connectivism, constructivism, authentic learning and experimental learning (Aguayo et al. 2018).

The principal outcome of the four studies demonstrated that DBR allowed for the design of new theory informed solutions by the implementation of IVR simulation underpinned by an educational need. The design further offered flexibility towards distance learning (Birt et al. 2017) and improved outcomes (Cowling and Birt 2018). Cochrane et al. (2020) further highlights high levels of student satisfaction (96%) and quality of learning (94%) for the 2017 iteration and 100% of participants found the IVR experience authentic and immersive within the 2018 iteration. Despite the promising results, most of the non-DBR reviewed focused on procedural performance and not a transferable educational need. Therefore, there is abundant room for further progress in determining the best approach to healthcare education learning design with further work required to validate the DBR process.

### Limitations

Limitations within this review are largely due to IVR simulation being an emerging and ever-changing field. This limitation is further evident when evaluating the impact of stress and the educational underpinning of the research. Study populations were mostly small and those investigated within a DBR methodology having little diversity as all were Paramedicine cohorts. This may be as a result of the search terms having a slight lean towards pre-hospital HCP’s.

The title review was not registered with PROSPERO as their criteria does not include ‘scoping reviews’ at present. Given the potential influence of the research team on the development, investigation, analysis and interpretation of the data, an independent reviewer was used at all stages of the analysis interpretation.

It may well be a factor that development and publication of IVR simulation literature has been hindered in recent years by the onset of the Covid-19 pandemic. This is a factor as many technology interventions require participants being in close proximity to both the equipment, researchers and within a closed room environment. With this, many universities have been closed or have yielded very high standards of safety regulations resulting in reduced research and publications in this area.

Finally, whilst cost, motion sickness, realism and haptic feedback are all important considerations, when exploring the impact of IVR simulation, an in-depth review of these factors was deemed outside the scope of this review.

## Conclusion

Despite the significant role that simulation plays in clinical education, the education design models underpinning most IVR fall short within the literature. However, we have found a potential for such theoretical underpinnings within DBR that could be applied to many contexts and technology enhanced healthcare learning environments. IVR also has the ability to offer a powerful authentic experience that has been reported to be equivalent to a real life experience and further offers transferable concepts such as stress and anxiety. Our understanding of how to measure and assess IVR stress and anxiety is still in its infancy with a dearth of evidence regarding agreed standards and approach. Therefore there remains a need for specific and focused research to deepen our knowledge and understanding of the impact of stress and anxiety within IVR clinical simulation and factors that might mitigate this potential debilitating response.

The strength of this scoping review is the broad perspective that presents a landscape of literature in the domain of IVR simulation within healthcare. Notwithstanding the relatively limited sample, this work offers valuable insights into IVR clinical simulation, its relationship to stress and the education design models currently being utilised to develop these concepts. Finally, being present in a non-physical world has the potential to offer the HCP boundless opportunity to develop technical and non-technical authentic real-world skills. Because of this, the authors suggest considerably more work is required in this area to ensure IVR is not only safe but also add value to those who use it.

## Appendix 1: Search terms

**Table:** 

#1	Paramedic* OR “Emergency paramedic” OR “Paramedic person*” OR “Emergency medical technician*” OR EMT OR “Ambulance person*” OR “paramedic Student*” OR “Medical student” OR “Doctor” OR “Nurse” OR Healthcare professional” OR “medical*” OR “clinical*” OR “paramedic*” OR ambulance* OR EMS OR “Emergency medical service” OR “emergency medical*” OR “first responder*”
	AND
#2	“Virtual reality exposure therapy” OR “exposure therapy” OR “virtual reality education” OR “Augmented realit*” OR “VR” OR “AR” OR “Virtual world” OR “Virtual environment*” OR “Mixed realit*” OR “Immersive environment*” OR “Virtual space*” OR “virtual reality” OR “MMR” OR “XR” OR “immersive cave” OR “virtual*” Or “immersive learning” OR “cave” OR “immersiv*”
	AND
#3	“Simulation*” OR "Patient Simulation" OR “Simulation training” OR “High fidelity simulation training” OR “High fidelity simulation” OR “clinical simulation” OR “high fidelity clinical simulation” OR “high fidelity*” OR “manikin” OR “Laerdal” OR “mannequin” OR “high-fidelity”
	AND
#4	Stress* OR Anxiet* OR “biological stress*” OR “Cognitive reserve*” OR “Mental process*” OR “brain reserve” OR “Cognitive Behavioral Therapy” OR “Cognitive Behavioural Therapy” OR "Performance Anxiety" OR "Test Anxiety Scale" OR "Manifest Anxiety Scale" OR "Test Anxiety" OR “cognitive*” OR “emotion*” OR “biometric*”
	AND
#5	“DBR” OR “EDR” OR “design based research” OR “design-based-research” OR “educational design research” OR “educational-design-research” OR “pedagogy” OR “andragogy” OR “heutagogy” OR “self-determined learning” OR “self-regulated learning” OR “self determined learning” OR “self regulated learning” OR “clinical education” OR “medical education”
**(OVID format)**	
#1	Paramedic OR Emergency paramedic OR Paramedic person OR Emergency medical technician OR EMT OR Ambulance person OR paramedic student OR Medical student OR Doctor OR Nurse OR Healthcare professional OR medical OR clinical OR paramedic OR ambulance OR EMS OR Emergency medical service OR emergency medical OR first responder
	AND
#2	Virtual reality exposure therapy OR exposure therapy OR virtual reality education OR Augmented reality OR VR OR AR OR Virtual world OR Virtual environment OR Mixed reality OR Immersive environment OR Virtual space OR virtual reality OR MMR OR XR OR immersive cave OR virtual Or immersive learning OR cave OR immersive
	AND
#3	Simulation OR Patient Simulation OR Simulation training OR High fidelity simulation training OR High fidelity simulation OR clinical simulation OR high fidelity clinical simulation OR high fidelity OR high-fidelity OR manikin OR Laerdal OR mannequin
	AND
#4	Stress OR Anxiety OR biological stress OR Cognitive reserve OR Mental process OR brain reserve OR Cognitive Behavioral Therapy OR Cognitive Behavioural Therapy OR Performance Anxiety OR Test Anxiety Scale OR Manifest Anxiety Scale OR Test Anxiety OR cognitive OR emotion OR biometric
	AND
#5	DBR OR EDR OR design based research OR design-based-research OR educational design research OR educational-design-research OR pedagogy OR andragogy OR heutagogy OR self-determined learning OR self-regulated learning OR self determined learning OR self regulated learning OR clinical education OR medical education

## Appendix 2: search results and strategy

Ovid ERIC (Abstract: full text/English/human)

1. Health care professional {including related terms}/ (59049 hits)
2. Virtual reality {including related terms}/ (1986 hits)
3. Clinical simulation {including related terms}/ (12237 hits)
4. Stress {including related terms}/ (13391 hits)
5. Education {including related terms}/ (4472 hits)
6. 1 and 2 /(11 hits)
7. 3 and 4 /(84 hits)
8. 1 and 5 / (599 hits)
9. 3 and 5 / (31 hits)
10. 1 and 2 and 3 and 4/ (5 hits)
11. 1 and 2 and 3 and 4 and 5/ (0 hits)

Ovid AMED (Abstract: full text/English/human)

1. Health care professional {including related terms}/ (7036 hits)
2. Virtual reality {including related terms}/ (155 hits)
3. Clinical simulation {including related terms}/ (101 hits)
4. Stress {including related terms}/ (3397 hits)
5. Education {including related terms}/ (191 hits)
6. 1 and 2 /(42 hits)
7. 3 and 4 /(14 hits)
8. 1 and 5 / (178 hits)
9. 3 and 5 / (5 hits)
10. 1 and 2 and 3 and 4/ (0 hits)
11. 1 and 2 and 3 and 4 and 5/ (0 hits)

Ovid PsychINFO (Abstract: full text/English/human)

1. Health care professional{including related terms}/ (99757 hits)
2. Virtual reality {including related terms}/ (2364 hits)
3. Clinical simulation {including related terms}/ (2764 hits)
4. Stress {including related terms}/ (91702 hits)
5. Education {including related terms}/ (1984 hits)
6. 1 and 2 /(500 hits)
7. 3 and 4 /(387 hits)
8. 1 and 5 / (1647 hits)
9. 3 and 5 / (41 hits)
10. 1 and 2 and 3 and 4/ (6 hits)
11. 1 and 2 and 3 and 4 and 5/ (2 hits)

EBSCO CINAHL complete, EBSCO MedLine (Abstract: full text/English/human)

1. Health care professional{including related terms}/ (98591 hits)
2. Virtual reality {including related terms}/ (183600 hits)
3. Clinical simulation {including related terms}/ (317807 hits)
4. Stress {including related terms}/ (1535686 hits)
5. Education {including related terms}/ (38474 hits)
6. 1 and 2 /(1533 hits)
7. 3 and 4 /(17554 hits)
8. 1 and 5 / (33607 hits)
9. 3 and 5 / (2108 hits)
10. 1 and 2 and 3 and 4/ (31 hits)
11. 1 and 2 and 3 and 4 and 5/ (17 hits)

SCOPUS (Abstract: full text/English/human)

1. Health care professional{including related terms}/ (172989)
2. Virtual reality {including related terms}/ (1936022)
3. Clinical simulation {including related terms}/ (4598825)
4. Stress {including related terms}/ (7018726)
5. Education {including related terms}/ (532591)
6. 1 and 2 /(2949)
7. 3 and 4 /(98204)
8. 1 and 5 / (28793)
9. 3 and 5 / (2093)
10. 1 and 2 and 3 and 4/ (43)
11. 1 and 2 and 3 and 4 and 5/ (17 hits)

Web of Science (Abstract: full text/English/human)

1. Health care professional{including related terms}/ (100025 hits)
2. Virtual reality {including related terms}/ (445991 hits)
3. Clinical simulation {including related terms}/ (1929266 hits)
4. Stress {including related terms}/ (3477910 hits)
5. Education {including related terms}/ (96497 hits)
6. 1 and 2 /(3800 hits)
7. 3 and 4 /(129380 hits)
8. 1 and 5 / (38042 hits)
9. 3 and 5 / (4178 hits)
10. 1 and 2 and 3 and 4/ (90 hits)
11. 1 and 2 and 3 and 4 and 5/ (71 hits)

## Appendix 3: characteristics of included articles

**Table:** 

Authors	Title	Publication year	Country	Journal	Sample size	Design
Aguayo, C., Dañobeitia, C., Cochrane, T., Aiello, S., Cook, S., & Cuevas, A	Embodied reports in paramedicine mixed reality learning	2018	New Zealand	Research in learning technology	32/21	Mixed method prototype
Birt, J., Moore, E., & Cowling, M	Improving paramedic distance education through mobile mixed reality simulation	2017	Australia	Australasian journal of educational technology	159	Experimental observation
Chang, T. P., Beshay, Y., Hollinger, T., & Sherman, J. M	Comparisons of Stress Physiology of Providers in Real-Life Resuscitations and Virtual Reality-Simulated Resuscitations	2019	United States of America	The journal of the society for simulation in healthcare	16	Pilot experimental
Cochrane, T., Aiello, S., Cook, S., Aguayo, C., & Wilkinson, N	MESH360: A Framework for Designing MMR-Enhanced Clinical Simulations	2020	New Zealand	Research in learning technology	35/30	Mixed methods
Concannon, B. J., Esmail, S., & Roberts, M. R	Immersive virtual reality for the reduction of state anxiety in clinical interview exams: Prospective cohort study	2020	Canada	Journal of medical internet research (JMIR)	49	Prospective, experimental, non-randomized controlled trial
Cowling, M., & Birt, J	Pedagogy before technology: A design-based research approach to enhancing skills development in paramedic science using mixed reality	2018	Australia	Multidisciplinary digital publishing institute (MDPI); information	159	Experimental design
Johnsen, K., Dickerson, R., Raij, A., Lok, B., Jackson, J., Shin, M., … & Lind, D. S	Experiences in using immersive virtual Reality Characters to Educate Medical Communication Skills	2005	United States of America	Institute of electrical and electronics engineers	7	Case study: mixed methods
Lerner, D., Mohr, S., Schild, J., Göring, M., & Luiz, T	An Immersive Multi-User Virtual Reality for Emergency Simulation Training: Usability Study	2020	Germany	Journal of medical internet research (JMIR)	18	Feasibility study: cross-sectional, one-group pre-test/post-test design
Price, M. F., Tortosa, D. E., Fernandez-Pacheco, A. N., Alonso, N. P., Madrigal, J. J. C., Melendreras-Ruiz, R., … & Rodriguez, L. J	Comparative study of a simulated incident with multiple victims and immersive virtual reality	2018	Spain	Nurse education today	35	Experimental observation: Comparative study
Rushton, M. A., Drumm, I. A., Campion, S. P., & O'Hare, J. J	The Use of Immersive and Virtual Reality Technologies to Enable Nursing Students to Experience Scenario-Based, Basic Life Support Training-Exploring the Impact on Confidence and Skills	2020	United Kingdom	Computers, informatics, nursing (CIN)	209	Mixed methods
Servotte, J. C., Goosse, M., Campbell, S. H., Dardenne, N., Pilote, B., Simoneau, I. L., … & Ghuysen, A. (2020)	Virtual Reality Experience: Immersion, Sense of Presence, and Cybersickness	2020	Belgium	Clinical simulation in nursing	61	Quasi-experimental
Wier, G. S., Tree, R., & Nusr, R	Training Effectiveness of a Wide Area Virtual Environment in Medical Simulation	2017	United States of America	The journal of the society for simulation in Healthcare	470	Comparative prospective cohort study

## Appendix 4: Results of individual sources of evidence

**Table:** 

Author and publication year	Title	Population	Context	Concept	Sub-Question 1	Sub-Question 2	Context	Outcome/Findings
		Healthcare Professional	Clinical Simulation	Immersive Technology	Stress measurement	Authentic Learning Design		
Aguayo et al. (2018)	Embodied reports in paramedicine mixed reality learning	Para student	Manikin Simulation	Mixed reality: VR + Cave	Questionnaire, SC, expression pedal, HR	DBR: blended	DBR/biometrics and simulation design prototype. DBR loop project for Mixed Reality simulation?	Emotional engagement, based on the subjective experience and differential biological responses
Birt et al. (2017)	Improving paramedic distance education through mobile mixed reality simulation	Para	Augmented Skill / avatar	Mixed reality	x	DBR: blended	Demonstrated distance education skill development with mobile mixed reality tool	Significant improvement for students who received the tools before residential schol
Chang et al. (2019)	Comparisons of Stress Physiology of Providers in Real-Life Resuscitations and Virtual Reality-Simulated Resuscitations	Doctor	Human vs Virtual simulation	VR augmented	HR (Hexoskin suit), Cortisol, NASA Task Load Index	x	Determine whether stress pathology changes were equivalent between VR and hospital resuscitations	Virtual reality resuscitations increase HR but show less stress physiology changes than ED resuscitations
Cochrane et al. (2020)	MESH360: A Framework for Designing MMR-Enhanced Clinical Simulations	Para student	Manikin Simulation	360 VR and Cave	HR, SC, Questionnaire	DBR: blended	Prototype iterations of a design-based research project. Triangulation of biometric and subjective feedback data	Highlights the development of implementation and data triangulation methodologies for VR clinical simulation education
Concannon et al. (2020)	Immersive virtual reality for the reduction of state anxiety in clinical interview exams: Prospective cohort study	OT student	Virtual simulation / avatars	VR augmented	STAI Y1, Test anxiety inventory	x	Effectiveness of anxiety reduction for OT students when using immersive VR	This investigation shows evidence of immersive VR’s capability to reduce state anxiety in OT students preparing for clinical practical exams
Cowling and Birt (2018)	Pedagogy before technology: A design-based research approach to enhancing skills development in paramedic science using mixed reality	Para student	Augmented Skill / avatar	VR/MR augmented	x	DBR	2 loop DBR project for mixed reality simulation of upper airway blockage	Improved outcomes and student enjoyment with an iterative approach to the problem
Johnsen et al. (2005)	Experiences in using immersive virtual Reality Characters to Educate Medical Communication Skills	Medical student	Augmented Skill / avatar	Cave design virtual characters	x	x	Impact and reaction of projector-based immersion on cognition and communication skills	Life sized virtual characters and Immersion contributed to the teaching/learning experience
Lerner et al. (2020)	An Immersive Multi-User Virtual Reality for Emergency Simulation Training: Usability Study	Doctor	Virtual simulation / avatars	VR augmented	x	x	Insights into the training effectiveness and media factors influencing learning and training in VR	Participants rated the VR simulation training positive in terms of training effectiveness and quality of the training execution
Price et al. (2018)	Comparative study of a simulated incident with multiple victims and immersive virtual reality	Student nurse	Human vs Virtual simulation	VR/360	Salivary α-amylase, BP, HR	x	Stress implications and efficiency of VR triage versus clinical triage for mass casualty incident	Virtual reality method is as efficient as (actor) clinical simulation for training on the execution of basic triage
Servotte et al. (2020)	Virtual Reality Experience: Immersion, Sense of Presence, and Cybersickness	Student nurse	Virtual simulation / avatars	VR augmented	Questionnaire, VAS, Mental Readiness Form	x	Investigation into immersion and sense of presence with a VR mass casualty incident	Immersive simulation induced a high level of sense of presence and a low level of cybersickness
Rushton et al. (2020)	The Use of Immersive and Virtual Reality Technologies to Enable Nursing Students to Experience Scenario-Based, Basic Life Support Training-Exploring the Impact on Confidence and Skills	Student nurse	Manikin Simulation	Immersive cave, 3 wall/8 wall and floor, shutter glasses	Confidence tool	x	To investigate confidence and competence in scenario based VR CPR training environments	Placing students in an unfamiliar environment influences the confidence and skills
Wier et al. (2017)	Training Effectiveness of a Wide Area Virtual Environment in Medical Simulation	Army medic, doctor, nurse	Manikin Simulation	Immersive Cave: sound, smell	x	x	Compare and measure performance, realism and satisfaction within an immersive environment	Exposure to stressful authentic environment should lead to improved performance and better patient outcomes. Expensive

*VR* Virtual reality: *Para* Paramedic: *SC* Skin conductance: *HR* Heart rate: *DBR* Design based research: *ED* Emergency department: *OT* Occupational therapy: 360 360 degree (technology): *BP* Blood pressure: *VAS* Visual analogue scale: *CPR* Cardiopulmonary resuscitation

## Data Availability

All data generated or analysed during this study are included in this published article.
